# Effects of the Digital Transformation: Qualitative Study on the Disturbances and Limitations of Using Video Visits in Outpatient Care

**DOI:** 10.2196/jmir.9866

**Published:** 2018-06-27

**Authors:** Linda Sturesson, Kristina Groth

**Affiliations:** ^1^ Department of Learning, Informatics, Management and Ethics Karolinska Institutet Stockholm Sweden; ^2^ Centre for Innovation Karolinska University Hospital Stockholm Sweden; ^3^ Department of Clinical Science, Intervention and Technology Karolinska Institutet Stockholm Sweden

**Keywords:** Video visit, outpatient care, disturbance, perceived limitation, telemedicine, telehealth, ethnography

## Abstract

**Background:**

Video mediated meetings with patients were introduced in outpatient care at a hospital in Sweden. New behaviours and tasks emerged due to changes of roles, work processes and responsibilities. The study investigates effects of digital transformation, in this case how video visits in outpatient care change work processes and introduces new tasks, in order to further improve the concept of video visits.

**Objective:**

Through real-time, social interactional features of preparing for and conducting video visits, the study examines clinicians’ perceived limitations and disturbances, and how the conditions between patients and clinicians may change when using video visits instead of face-to-face meetings in outpatient care.

**Methods:**

Qualitative methods have been used including 14 observations of video visits at two different clinics and 14 followup interviews with clinicians. Transcriptions of interviews and field notes were thematically analysed, discussed and synthesised into themes.

**Results:**

Disturbances and limitations related to the technology were related to time; a flexibility to schedule the meeting unbound of place, frustrations when the other part was late for the scheduled meeting, and that more experienced users of video visits usually waited longer before logging in. They were also related to sound; problems getting the sound to work satisfactory during the video visits, and problems with the image. Disturbances and limitations related to the surroundings were related to both the patient’s and the clinician’s environment; the principle of video technology in itself may affect the experience and the content of the consultation, and the surrounding chosen changes the conditions for and reduces the participants’ field of view.

**Conclusions:**

We could see 1) a transformation of roles and responsibilities when turning from face-to-face meetings to video visits, 2) that video visits add new circumstances, with a risk of introducing disturbances and limitations, that in turn affects the content of the meeting, 3) that avoiding negative disturbances during a video visit, requires a sensibility from the clinician’s side as well as a trust in the patient’s judgement, 4) that both expected and unexpected disturbances and limitations during a video visit affect the clinician’s behaviour, feelings, the content of the meeting and how the clinician’s relate to the different components of the concept, and 5) that there is a change of roles introduced when conducting video visits, eg, the clinician taking the first line support if both (s)he and the patient encounter problems with the technology.

## Introduction

Over the last few decades, telemedicine has evolved as a solution for the healthcare sector to meet the challenges of an increasing patient population [1,2]. One telemedicine solution is video-mediated meetings or consultations (hereafter called video visits) between patients (including relatives) and clinicians. Video visits provide a new way of conducting clinical meetings, potentially reducing costs and providing services for patients living in remote or rural areas [1,3]. There is no doubt that video visits can be beneficial for different stakeholders from different perspectives [1,3], but the implementation of telemedicine also causes changes in the organization of work; tasks and processes; and identities, roles, and authority, potentially affecting the power relationships among the participants; and participants’ expectations of the meeting (cf [4-7]). Recent research has shown that video visits in outpatient care settings appear to be safe, effective, and convenient; however, there are complex challenges related to their adoption by clinicians [8]. Implementation of new technology in consultations can be met with skepticism by clinicians. Some view technology as something that interferes with good clinical practice and the exercise of professional judgment [1]. In comparison to home visits, video visits carry advantages for clinicians, such as being cost- and time-efficient [9]. Satisfaction with video technology has been explored in surveys [9], but problems with technology are seldom explored in-depth [1]. Experiences regarding the implementation of video visits have not been examined very well [3], but recent research addresses the question of implementation and has generated five key recommendations concerning how technology should be introduced: iterative introduction with the involvement of staff, time for reflection with staff and patients, relations with the information and communication technology (ICT) department to establish roles and processes, understanding patients’ conditions, and flexibility of use to fit patients’ needs [8]. If video visits are going to be beneficial at the macro level, they need to attract users (ie, clinicians and patients) at the micro level by considering professional dimensions and meeting the users’ needs. Hence, the users’ interplay, expectations, experiences, and perceived benefits should be accounted for during concept development.

The technology used, together with its principles and procedures employed when conducting video visits, can produce limitations and add disturbances that may, positively or negatively, affect how the meeting is conducted and its outcome. A video visit implies a geographical separation between clinician and patient (cf [10]) with a new location added to the healthcare consultation. Introducing non-clinical settings may affect those involved, the consultation, and the outcome of the meeting because a healthcare environment manifests social orders [5]. Also, medical spaces and physical settings facilitate the maintenance of professional and patient roles. Hence, video visits introduce new spaces that may challenge or reinforce the established performances, relations, and hierarchies [5]. Petersson [11] highlighted how telehealth “allows health professionals to unfold new spaces of visibility,” since it provides a link between the patient’s setting and healthcare institution. However, research on telemedicine seldom includes the place, as it appears to be considered irrelevant in the discourse of telemedicine [10]. The impact of the patient’s environment and surroundings, when conducting video visits, appears to have been less thoroughly explored, even though there is evidence that the place matters [10-13]. For example, even though the complex communication of a video visit can be affected by the environment [13], the video visit concept seldom includes recommendations for the physical environment. Other aspects however are considered, such as recommendations that everyone present during a video consultation should be identified. This is because otherwise sensitive information could be disclosed to individuals not in the field of view of the webcam [12]. Privacy considerations are generally handled by the clinician during a physical meeting, but it might be more difficult when the patient is located in another place due to the limited view of the camera. What happens in the patient’s environment and surroundings may cause disturbances and create limitations, which can sometimes be easily resolved [8], but may also affect the outcome of the consultation. It is well known that the physical environment affects patients’ satisfaction levels, attitudes, and work performances during the meeting [14,15]. When the space of care is no longer shared physically, and the connection between the clinician and patient is mediated by technology, new questions arise: What might take place that cannot be seen? What disturbances and limitations may occur? What effects can such disturbances and limitations cause?

The aim of this study was to explore the disturbances and limitations experienced by clinicians when conducting video visits and how these disturbances and limitations affected roles, content, and perceptions. We explored what happened when the space of care was spatially shared between two environments and mediated by video, with the overarching goal of improving the concept of video visits, thereby meeting the need for a more efficient care. The study was conducted in an outpatient care center at a university hospital in Sweden.

## Methods

The study was qualitative and explorative in its approach. Interviews with clinicians and observations of video visits were conducted to generate data. The focus was on the situatedness in the use of video visits and situated actions when clinicians conducted such visits. Additionally, informal and formal settings in everyday work and ad hoc individual conversations related to video visits were observed and used to understand the phenomenon of video visits and their role in a wider context.

### Approach to the Research Area

Theoretical perspectives in symbolic interactionism provided a source of inspiration and a starting point, with frameworks suitable for analyzing the social reality and understanding human behavior and human feelings. The social interaction can be influenced by moods, weather, locations, and environments. The individual defines the situation both consciously and unconsciously and human behavior is seen in relation to the whole context [16]. Diversity, as well as commonalities, are sought with an open mind, with attention given “to what falls out of view or falls between the cracks” [17]. In our study, the video visits were part of a treatment program that included several consecutive meetings. The consultation is a social interaction between a clinician and a patient and/or relative, where at least one of them has a predetermined goal for the meeting. What happens among those involved can be understood as social acting and, more specifically, as an instrumental or planned action. For example, a clinician may have the goal of discovering the patient’s behavior since the last meeting, progress, or side effects, etc. To achieve this goal, the clinician will prepare by reading the patient’s medical record and making notes on what to address during the consultation. However, each consultation session is a link in a longer treatment chain—a path where each situation affects the outcome of each session (cf [18]).

Clinicians develop skills based on physical consultations, and their face-to-face visits become the norm for clinical meetings [7]. The clinicians’ frame of reference is, thus, the traditional physical meeting or a follow-up by phone. When introducing video visits, clinicians are therefore likely to compare video visits to traditional clinical meetings. Anything that is perceived as a deviation from the norm can be understood as a disturbance. A disturbance is defined as something that differs from the norm, from what clinicians are used to, and can be perceived as negative or positive. Disturbances can cause perceived limitations, which can be seen as disadvantages or advantages. In our study, we defined a limitation as an abstract feeling or experience of something not being enough or being a restriction. For example, surroundings or situations can serve as limitations. Limitations can lead to disturbances and disturbances can lead to limitations; there is an interplay between the two concepts. Disturbances and limitations can be unexpected in some cases and planned for in others. The technology, procedure, and principles of video visits can cause both disturbances and limitations.

In our study, we explored video visits by gathering examples of disturbances, both negative and positive, and limitations (as perceived by clinicians, not by patients/relatives). We examined what gave rise to the disturbance, how the disturbance was interpreted, and its consequences. This includes understanding the video visits as present situations happening in a context including space and place, even though the participants are located at a distance from each other.

### Ethical Approval and Consent

Ethical approval for the study was given by the Regional Ethical Review Board in Stockholm before data gathering (reference number: 2016/1027-31). The clinicians obtained written informed consent for participation and for publication (including information about participation, anonymity, purpose and objectives of the study, and responsible researcher) from patients, relatives, and guardians. Participants were offered video visits instead of physical meetings. The consent form was either sent by email or given by hand to the patient/relatives. The clinicians signed a written consent for participation following review by the researcher.

### Context

Two patient flows were involved in this study, named Clinic A and Clinic B. Both clinics treated patients with obesity. The clinics had congruent goals, agendas, and philosophies for their treatment. The content of care was mainly based on a humanistic perspective of health and disease, with lesser focus on biomedical data such as weight and body composition. However, these variables were still used as treatment outcome assessments. Video visits at the clinics were part of a treatment program that included several consecutive meetings aimed at helping patients to successfully implement lifestyle changes. Clinicians supported patients in their efforts to achieve behavioral and lifestyle changes. Between visits, patients were asked to work actively on lifestyle changes by themselves. Both clinics shared the same view about using video visits as complements to face-to-face visits and for follow-ups. The staff consisted of doctors, nurses, psychologists, nutritionists, occupational therapists, and physiotherapists. Clinicians at Clinic B also had competence in cognitive behavior therapy. Differences between the clinics are described in Table 1.

**Table 1:** Comparison of patient population, implementation stages, and settings of Clinic A and Clinic B.

Aspect of the setting	Clinic A		Clinic B
Patient population	Children and adolescents with obesity (2–18 years old)	Adults with obesity (>18 years)	
Responsible for and involved in the treatment	Relatives were responsible for and provided an important role in the treatment. Relatives of young children visited the clinic together with the child. Follow-ups and reconciliations were made over phone with relatives of young children and not with the child. Teenage patients were assessed by the clinicians to decide if they were mature enough to take responsibility for their own treatment. If so, the relative usually did not participate in follow-ups.	Patients were responsible for their own treatment. Relatives were not present during meetings.	
Stages of implementation of video visits	Video visits began when the research study started.	6-month history of carrying out video visits	
Setting for video visits	One room was used for video visits. The room was equipped with a computer, camera, and headset. Clinicians booked the room before the video visits.	The clinicians used their own room, with their computer equipped with camera and a headset.	

**Table 2:** Number of observations and interviews conducted at Clinic A and Clinic B, with clinicians, patients, relatives, or both patients and relatives.

Method	Total	Clinic A					Clinic B		
		Total	Clinician	Patient	Relative	Both	Total	Clinician	Patient
Observation	13	9	6	5	3	1	4	2	4
Interview	14	10	6	—^a^	—	—	4	2	—

^a^Patients or relatives were not interviewed.

### Technology and Devices

The concept used for the video visits was developed for less complex meetings in outpatient care. The technology included an ordinary video conferencing tool with encrypted communication, capable of producing adequate quality for seeing and hearing each other and for sharing documents. The technology could not be used to connect sensors used for monitoring parameters, and the quality of the video was not high enough to provide details of, for example, skin issues. A complex video visit, such as when a neurologist needs to see small detailed movements during care for patients with Parkinson’s disease or demonstrate exercises to a patient [19], may require equipment of higher quality, as well as additional space in front of the video camera for specific exercises. The patient/relative typically used his or her own device, such as a computer, mobile phone, or tablet, with a webcam, speaker, internet connection and web browser, or the video conferencing app.

### Respondents and Recruitment

In preparation for the study, two clinics were selected to participate. They were identified from the second author’s work of introducing video visits in outpatient care settings at hospitals. One clinic was selected because it had successfully adopted the concept of video visits earlier in the year. The other clinic was selected because it had shown interest and carried out test video visits, but had not yet started. Two clinicians from the first clinic, already conducting video visits, and six clinicians from the second clinic, who wanted to start video visits, agreed to participate in the study.

At both clinics, the staff selected patients or relatives for video visits. Video visits were only offered to patients who had physically presented to the clinic at the beginning of their treatment. The clinicians offered video visits to the selected patients either during a physical meeting or through a telephone contact. The patients had the opportunity to accept or decline video visits. During the study period, there were patients who declined. The clinicians who conducted the video visits had previously met face-to-face with the patients. Selecting patients for video visits and the criteria used in the process have been described in a paper sent for publication (Sturesson and Groth, in preparation).

If patients accepted a video visit, the clinicians asked them if they wanted to participate in the research study. The question was asked of the patient/relative during a face-to-face meeting, a phone call, or a previous video visit (at Clinic B, where video visits were used before the research study started). The staff, patients, and any guardian provided written informed consent to participate in the research.

### Data Collection

The data collection consisted of a total of 13 observations and 14 interviews; see Table 2 for more details. Six of the clinicians conducted two video visits each and were, therefore, observed and interviewed twice. However, one of the interviews was conducted without an observation (see below), resulting in a total of 13 observations and 14 interviews.

Each observation started before the actual video visit and included the time for the clinician’s immediate preparation. The researcher was located in the same room as the clinician and was visually and verbally presented to the patient/relative at the beginning of the video visit, giving each patient a chance to withdraw his or her consent. During the video visit, the researcher observed the meeting from a position out of sight of the webcam, that is, the patient/relative could not see the researcher. The observations were partly exploratory and partly structured. Some aspects, such as start and end time, patient’s location, and number of participants, were predetermined and noted in the observation protocol. These were combined with field diaries that contained the exploratory observation notes. The observations were not recorded, photographed, or filmed.

The interviews were in-depth, contextual, and semi-structured and were conducted with the clinicians after, and in addition to, each video visit. Of the 14 interviews, 13 were conducted face-to-face and one by phone. One of the interviews occurred without an observation, since the patient withdrew consent to participate in the study as the observation was about to start. The interview was still conducted after the video meeting. The interviews were recorded and transcribed verbatim.

In addition, the researcher attended formal encounters (eg, treatment conferences with clinicians) as a passive observer and participated in informal gatherings (eg, lunches and other breaks), taking field notes to capture the clinical discourse and clinicians’ perceptions and thoughts about video visits and without interfering in the discussions taking place. All data were gathered during a contiguous period of 3 months during 2016.

### Analysis

The analysis process followed a qualitative approach [20], in which the transcripts of interviews and field notes were read through several times to familiarize with the content. During the reading, themes were identified and noted on a blank sheet. Corresponding transcripts and field notes were read iteratively to gain a full picture of the collected data. A conceptual framework was created. After this initial process, the transcripts of interviews and field notes were thematically analyzed [21]. Data were then read through again and coded to match the themes in the developed conceptual framework.

**Table 3:** Being late or being on time, based on observational data on each video visit.

Observation data	Total	Clinic A										Clinic B			
Video visit number	N/A^a^	1	2	3	4	5	6	7	8	9	10	11	12	13	14
The clinician was logged in on time	9	Y^b^	Y	Y	N^c^	Y	N	Y	N	N	Y	Y	N	Y	Y
The patient/relative was logged in when the clinician logged into the virtual meeting room.	3	N	N	N	N	N	N	N	N	Y	N	N	Y	N	Y
The clinician calls patient/relative	6	Y	N	Y	Y	N	Y	Y	N	N	Y	N	N	N	N
The video visit started at the scheduled time	4	Y	Y	N	N	Y	N	N	N	N	N	N	N	N	Y
The video visit started later than the scheduled time	10	N	N	Y	Y	N	Y	Y	Y	Y	Y	Y	Y	Y	N
Number of minutes after scheduled time that the video visit started	—^d^	—	—	12	26	—	23	19	9	3	28	3	3	6	—

^a^N/A: not applicable.

^b^Y=Yes.

^c^N=No.

^d^"—" indicates that the video visit started on time.

Spreadsheets were used to organize and sort the data. To find and keep track of patterns in the material, themes were separated into different rows in the spreadsheet, and each interview and the corresponding observation was sorted into different columns. Pieces of the text were sorted to the appropriate cells. The principle of spreadsheets was also used to analyze and find patterns in the quantitative data (such as the data described in Table 3). The data, themes, and sorting were continuously discussed throughout the analysis.

Video visit number 6 only consisted of an interview since the patient declined to participate in the study at the last minute. The interview was conducted immediately after the video visit.

The themes were synthesized into two overarching categories: “Selecting patients for video visits” and “Disturbances and limitations.” From the analysis, it became clear that selecting patients has added a new task for clinicians and that video meetings have introduced disturbances and limitations related to both the technology and surroundings. This paper focuses on the second category: Disturbances and limitations. The themes sorted under this category were issues of time and senses related to the technology used and issues of space and place related to the surroundings used by the clinicians and patients/relatives.

In the Results section, quotes are used to illustrate situations causing a disturbance or where a limitation was identified. The quotes chosen represent situations that occurred once or several times, illustrating an effect of something that may occur in other situations. When illustrating a situation related to the theme with excerpts from the data, we use the notation Clinic X, Int_Y, or Obs_Y, where Int stands for interview and Obs for observation. The interview that followed an observation of a video visit was given the same number as the observation, that is, the number of the video visit.

## Results

### Overview

We have identified several situations wherein clinicians experienced disturbances and limitations when conducting video visits. Disturbances and limitations were related either to the technology or surroundings.

As described above, we defined a disturbance as something that differed from the norm and from what the clinicians were used to. Disturbances can cause perceived limitations and can be perceived as negative but also, on occasion, as positive by participants. In addition, the technology and principles of video visits occasionally caused limitations. As pointed out in the Methods, we defined limitations as an abstract feeling or experience of something not being enough or as something being restricted due to, for example, the surroundings or situation.

### Disturbances and Limitations Due to the Technology

We identified disturbances and limitations related directly or indirectly to the technology used during video visits, which are presented based on two themes: *Time* and *Senses*. The time-related aspects presented below are *Flexibility in Time and Space*, *Being Late and Being on Time*, and *Waiting and Logging in*. Aspects of senses presented below are *Hearing and Being Heard* and *Seeing and Being Seen*.

#### Time: Flexibility in Time and Space

Clinicians at both clinics saw advantages related to flexibility in time when using video visits. Video visits made it possible for those involved to schedule the meeting unbound from place, eg, if the patient lived or worked far from the clinic. Clinicians who were more experienced in using video visits also saw benefits for themselves. For example, some were able to redistribute their working hours by working longer one day, as they no longer depended on the clinic’s regular business hours or the number of staff present at the clinic. According to safety regulations, at least three members of the staff needed to be present in the office when a clinician physically met with a patient. In turn, working longer one day made it possible for some clinicians to go home earlier another day. This flexibility in time could be seen as a positive disturbance.

#### Time: Being Late and Being on Time

During video visits, we observed several examples of worries and frustrations when the clinician or patient was late for the scheduled meeting. Table 3 specifies how many video visits started at the scheduled time and, if not, how many minutes late the visit started, who logged in on time, and whether the clinician called the patient.

Clinicians at Clinic B did not call patients who were late, whereas clinicians at Clinic A called the patients during six of the observations. The clinicians waited up to 7 minutes before calling if the patient was late. The delays observed were caused by (time) planning aspects, external circumstances, and technical issues. The clinicians always sent the number and code for the web-based video room to the patient before the meeting took place, but the routine differed among the clinicians with regard to how far in advance they sent the code in relation to the scheduled meeting. Some sent the information when the meeting was booked, and some sent it the same day as the meeting. The patients learned their clinicians’ routines.

In one of our observations, the clinician sent the information to the patient several hours later than usual. The information was actually sent after the meeting was scheduled to start. This caused the patient to become worried and confused about the time of the video visit (Clinic B, Obs_12). When a patient physically arrives at a clinic, he or she reports to the reception. This may give the patient a sense of security about not being forgotten. In another of our observations, the video visit started 20 minutes late because a previous face-to-face meeting ended later than planned. This was distressing to the clinician, who did not see any opportunity to notify the subsequent patient of the unexpected delay. When the meeting started, the clinician perceived frustration from the patient and relative. Further, the patient had plans after the meeting, leading to a shorter meeting than that for which the clinician had planned (Clinic A, Int_6 and Obs_6).

Being late due to external circumstances could also be related to the weather or to technical problems:

One of the clinicians at Clinic A arrived at the clinic 5 minutes before the video visit was scheduled to start because of heavy snowfall causing problems in the morning traffic. The clinician started to read the patient journal. Nine minutes after the scheduled time, the clinician entered the room dedicated for video visits, logged into the web-based video room, and noticed that the patient has not yet logged in. The clinician wanted to call the patient but there was no contact number with the hospital’s central server on the computer, and the patient’s phone number could not, therefore, be reached. Instead, the clinician had to take out the Siths card from the computer, walk back to her office to find the patient’s contact details on her own computer, and then go back to the video visit room and log in to the computer again. The clinician called the patient, who had still not logged in. It turns out that they were logged into different web-based video rooms, something that, in principle, should not be possible. After 23 minutes, they managed to connect. [Clinic A, Obs_4]

In this case, the meeting started late because the clinician was late and also because the patient was logged into the wrong meeting room. These delays caused a ripple effect of delays, and thus, frustration and stress were experienced by all participants. Valuable time was lost and the meeting was shorter than planned.

#### Time: Waiting and Logging In

If they were not delayed, the clinicians at Clinic A usually logged into the web-based video room approximately 4–10 minutes before the meeting was scheduled to start. The clinicians who were more experienced at using video visits (at Clinic B) usually waited longer before logging into the web-based video room but also gave the following, somewhat contradictory, explanation for logging in 2–5 minutes in advance:

If I log in 5 minutes in advance […] then I may have replied to an email or so, but also 2 minutes, that is too short a time to start with something, and then you just sit there and look at the screen, which feels a bit meaningless.

Clinic B, Int_11

The same clinician noted in the interview that she became restless while sitting and waiting for the video visit to start. She likened it to waiting for a bus and stated that she starts to think about other things and then suddenly realizes that the patient has logged in. Another clinician, from the same clinic, reported similar reflections. She reportedly felt stressed if she logged in too early because she then had difficulties doing other tasks, since she needed to keep an eye on the screen to be able to see when the patient logged in (Clinic B, Int_13).

Another clinician reflected that, if a patient was delayed for a face-to-face meeting, then they could do other things because they were in their own office and could be notified by reception when the patient arrived. In a video visit, the clinician had to actively check the screen now and then while waiting (Clinic A, Int_3).

Hence, video visits introduced limitations related to time management while waiting for the patient to log in. One strategy clinicians at Clinic B developed to overcome this was to log in as late as possible.

#### Senses: Hearing and Being Heard

On several occasions, there were problems getting the sound to work satisfactorily during video visits, affecting both the clinicians and the video visit itself. Problems with sound affected communication in that clinicians had to repeat themselves and ask “*what*?” more often. In some cases, system usability caused problems, in others, it was connectivity. In both cases, clinicians had to take on the role of first-line support, trying to figure out the cause of the problem: “The clinician asked if the patient has an icon for sound on the phone. The patient nodded and the clinician said: ‘try to push it […] I can see that the sound is not on, from your side” (Clinic A, Int_3).

Problems with the sound caused irritation: “it’s happened so many times that the sound hasn’t been working and it is a frustration” (Clinic A, Int_1). In one case, the clinician made a joke of it and blamed the bad weather and they then talked about a technical solution, discussing volume settings and differences between conducting video visits using a mobile phone and a laptop (Clinic B, Obs_13).

Problems with the technology not working as expected created barriers to the adoption of video visits. At the same time, when the sound did not work as expected, clinicians created situation-based solutions to these in situ problems. At Clinic A, clinicians would bring a phone to the video visit room: “The sound didn’t work and it’s happened once before […] but now I’ve solved this by having a phone with a speaker during the video visit” (Clinic A, Int_3). The in situ problem thus led to a pre-conceived notion of sound problems, for which the clinicians planned in advance. We observed that sound-related problems were shared with colleagues during breaks, resulting in clinicians who had never performed a video visit taking a phone when they began to carry out video visits.

#### Senses: Seeing and Being Seen

Video visits differed from phone consultations in that the clinician and patient could see each other. During video visits, the image was sometimes missing at the beginning of the visit or it sometimes disappeared during the visit for various reasons. This affected the clinician and the video visit in different ways.

One example illustrates how this could affect the clinician: “If the screen closes down all the time […] if you have poor connectivity, it might shut down like that and it can be a bit annoying because then maybe you’re talking about that instead ‘oh, now you’ve disappeared, but now you’re back’” (Clinic B, Int_13). In one case, the same clinician conducted a video visit and anticipated bad connectivity. She adapted the video visit to technical circumstances by planning the session’s content differently, knowing that visits like these tended to be “more choppy,” and she also lowered her own expectations; thus, the video visit became more “undemanding.”

Video problems could occur if the user was inactive on the computer, resulting in a locked screen mode, but with the web-based video meeting room still running. The clinician and the patient/relative could still hear each other but not see each other. For a clinician new to video visits, this could be disturbing, not knowing what happened or how to get back into the meeting. To avoid this, the experienced clinician moved the mouse quickly and entered the log in code, without affecting the meeting.

In one of our observations, a patient/relative suddenly disappeared from the screen but it was not related to being inactive. In this case, it may have been intentional (see example from Clinic A, Obs_1 under *the patient’s surrounding,* in the next section), but such a disturbance could also be caused by technology; for example, if the bandwidth goes down. Another reason for the patient/relative disappearing from sight involved the clinician sharing a presentation, eg, of a growth curve, or when a clinician opened a medical journal or calendar on the screen, thereby hiding the video conferencing window. This kind of planned disturbance could still cause inconveniences or confusion when the shared presentation was ended or the other windows were closed. The patient/relative could, if using a mobile phone or tablet, be in a different surrounding with other disturbances and limitations than before (Clinic A, Obs_9).

Technology caused limitations and disturbances that were perceived as negative from the clinician’s point of view. On the other hand, video visits also served as an extended eye for the clinician and were perceived as something positive since they provided the opportunity to see the patient’s context: their home, workplace, or school. This was expressed as something that provided more insight into the patient’s personality and the feeling of achieving a closer contact: “now I notice that I get a more personal picture of the person when I come into their home, because then suddenly it becomes real […] it becomes a closer contact somehow” (Clinic B, Int_13). The same clinician also said,

You can kind of take part in their private life. Then, whether it’s important, I can’t say. I think it can have a kind of effect for the compliance … maybe becomes positive when I say... what a nice color, and you see that everybody becomes happy. It’s really something personal, and it’s just small comments... I think it can be important for compliance, but I don’t know if it has any effect on the treatment.”

Clinic B, Int_13

The clinician experienced this differentially in comparison to face-to-face visits and perceived it as a positive aspect of video visits. The extent to which the patient saw this as a positive or negative disturbance or limitation was not stated.

### Disturbances and Limitations Due to the Surroundings

We identified disturbances and limitations related directly or indirectly to the surroundings used during video visits, which are presented based on the themes *the patient’s surroundings* and *the clinician’s surroundings*. The surroundings are related to both place and space.

#### The Patient’s Surroundings

Video visits imply that the patient and clinician are in geographically different locations. Using videos as a tool for communication brings a new field of view to the healthcare meeting: the patient’s/relative’s physical environment. When the clinician sees the patient in his or her environment and, at the same time, uses the sense of hearing, the patient’s/relative’s surroundings are brought to the meeting. What is happening in a specific situation, that can be both seen and not seen, is interpreted and affects both the clinician and patient, their actions, the content of the meeting, and its outcome.

Hence, the principle of video technology, which makes it possible to see and hear, may affect the experience and content of the consultation, as can be seen in the following example:

The clinician is conducting a video visit with the relative of a younger patient. After 10 minutes’ discussion, it becomes clear that the patient has gained a relatively large amount of weight since the last meeting. When the clinician asks “why?,” the relative said, “*I’ve no idea, don’t ask me.* ” They continued to talk about the weight gain, about what may have happened, and so on. Suddenly the screen turned black for a while and when it came back the audio on the phone was off (the phone was being used because of audio problems with the video connection). The clinician called the relative and they came back into the meeting, but soon, the screen again turned black, this time for a couple of minutes. When the clinician called back, the relative said they needed to end the meeting because the patient became sick and required care (Clinic A, Obs_1).

In the follow-up interview, the clinician said that she found it difficult to be sure whether it was a technical problem that caused the interruption or it was caused intentionally by the relative or the patient because of an unwillingness to discuss his or her recent weight gain. The clinician said that she found it peculiar that the patient suddenly became sick, but since she could neither see nor hear the patient during the video visit, she did not know what was going on in the patient’s surroundings (Clinic A, Int_1).

In another example, the patient’s surroundings provided a space that was unknown to the clinician other than that the patient would be in school: The video visit began and the patient, a teenager, was outside. From knowing that the patient would be in school, the clinician assumed the patient was in the schoolyard. The clinician felt that the patient had a roving eye, and thus assumed the patient “felt uncomfortable in the situation and uncertain; either [the patient] was scanning the environment […] or was just uncomfortable with the situation of sitting and looking into the camera” (Clinic A, Obs_8 and Int_8).

The clinician was not aware of whether there were other people in the surrounding area. However, the clinician’s assumption of a schoolyard may have affected the interpretation of the patient’s behavior, creating a sense of disturbance in the consultation: “I believe that [the patient] was perhaps a little bit stressed” (Clinic A, Int_8). As a result, the clinician did not ask further questions about sensitive subjects and “did not stay with every question as long as usual” (Clinic A, Int_8). Also, the clinician knew the patient was going back to class “so there was a time aspect as well, somehow the teacher was expecting him back.”

The environment may be a disturbing component of the meeting, in an interplay with what the clinicians can see, what they interpret about the unseen environment from the patient’s behavior, and time, all of which are bound to the patient’s environment. These three issues affected the actions of the clinician and limited the content of the consultation. In some cases, the clinician asked beforehand where the patient would be during the video visit. In one case, the patient answered the video call when in a locker room. The clinician asked if they might be disturbed, but the patient, a teenager, said the risk of disturbance was low. However, the patient and clinician agreed to use a code word if someone entered the locker room during the video visit, so they could close down the meeting without the person entering understanding the meeting’s content.

During one observation, the patient was in a parked car. Something happened during the video visit, something the clinician in the follow-up interview described as a “glitch,” which she interpreted as someone entering the car. Since the meeting time was almost over, the clinician ended it a little more quickly than she would have done if it had been a face-to-face meeting. The clinician noticed that she did not receive the same focus after the “glitch,” and she felt that the patient was indicating that the meeting time was almost over (Clinic A, Obs_2 and Int_2).

In another example, the patient, a child, was participating in a video visit at home when all of a sudden the child looked to the side several times and began to smile and laugh. The clinician asked the patient if there was another person who wanted to participate in the meeting. The patient said the younger sibling’s name (Clinic A, Obs_7). The clinician perceived this as a disturbance and said that the patient made signs to the sibling to send them out of the room:

I saw […] what happened there, talking about what you see and can’t see […] but then I wonder, [The relative] was clear with, ok now I’ll leave you […] you can sit in peace and quiet […], but now the sibling […] I could say that, the next time, maybe ask [the relative] […] to talk to [the sibling] and say that this is [the patients’] time so he doesn’t feel that [the sibling] is standing there and listening.

Clinic A, Int_7

These examples imply that the place itself may not only affect the meeting; rather, the meeting can be affected by events that occur in that place. Two of the interviews exemplified how the clinicians planned for this beforehand:

You can prepare when you book the video visit, [and ask the patient] which environment do you feel safe in […] where do you feel you can talk freely? Is it at home or […] a private room at the library or whatever?

Clinic A, Int_8

To minimize moments of distraction in the same way as if you were meeting here, you close the space around you so it’s only you and the patient. If he was sitting in a kitchen with younger siblings around, then I would recommend that he goes to a calm place where it’s easier for him to focus. Whether it’s his room or anywhere else, that doesn’t matter.

Clinic A, Int_7

#### The Clinician’s Surroundings

The clinicians at Clinic A all conducted video visits in one room dedicated to such visits, also used as a patient kitchen during face-to-face meetings. At Clinic B, the clinicians conducted video visits in their own rooms. This resulted in clinicians at Clinic A having to perform the extra task of double-checking the availability of the room before scheduling a video visit, something that was perceived as a disturbance or a barrier for video visits.

A video visit adds in the patient’s and/or relative’s environment and surroundings, but through the video technology used, it also changes the conditions and reduces the patients’ field of view. For clinicians at Clinic B, who were using their own rooms and were more experienced, insights about the field of view also affected their behavior. For example, we observed two cases in which clinicians brought coffee into the video visit. When asked about this, the clinician stated that this would never happen if the patient had visited face-to-face as that would not be professional. However, since the clinician knew that the coffee was not visible to the patient, it was perceived as acceptable to have it in the room during the video visit.

**Table 4:** Summary of findings.

Theme		Disturbances		Limitations	
**Time**					
	Flexibility in time and space		Unbound from place		Possibility to schedule meetings outside the opening hours of reception (Positive) Easier to schedule meetings with patients living far from the clinic (Positive)
	Being late and being on time		Frustration		Lack of functionality to communicate delays (Negative)
	Waiting and logging in		“Dead-time” when waiting for the meeting to start Need to actively check if the patient has logged in		Lack of functionality to be alerted when other parties are logged in (Negative)
**Senses**					
	Hearing and being heard		Technology problems Technology problems shared among colleagues		Barriers to adopting video visits (Negative) Adjust the meeting to known problems (Negative)
	Seeing and being seen		Being inactive on the computer Technology issues known beforehand Patient disappearing out of sight Extending the eye of the clinician into the patient’s context		Locked screen mode (Negative) Adjust the plans of the meeting (Negative) Difficult for clinician to understand the cause of this action (Negative) Gaining insight into the patient’s personality, adding a feeling of getting closer to the patient (Positive)
Patient’s surroundings		Patient disappearing out of sight Field of view changes Unknown spaces where disturbances are difficult to understand The environment itself and what can and cannot be seen		Lack of understanding about the cause of action (Negative) Adding the context of the patient (Positive) The content of the meeting (Negative)	
Clinician’s surroundings		The need to check the availability of room Decrease in the patient’s field of view		Barriers to adopting video visits (Negative) Only parts of the room needed to appear professional (Positive) Only visible clothing needed to be professional (Positive)	

Another example was when a clinician took off her shoes during a meeting (Clinic B, Obs_13). In the follow-up interview, the clinician stressed that this was not something that would happen in a face-to-face visit; then, she would always keep her shoes on. Also, one clinician said that she did not need to bother about a clean desk, as long as it was out of sight of the patient: “they don’t see my desk and that’s pretty nice, because then I can have it a little messier” (Clinic B, Int_11). She also noted not having to put private belongings somewhere else; a suitcase was standing in the middle of the room during the video visit and her coat was on a hanger. If a patient was visiting face-to-face, these objects would be hidden. This was seen as a part of being a professional, in the sense that patients should not be exposed to items from the clinician’s personal life including pictures, coats, and bags. Similarly, one of the clinicians at Clinic B said that when she used video visits, she used the opportunity to stand up at her (height adjustable) desk while using the computer. The awareness of the patient’s field of view and how the surroundings and behavior could change compared to a face-to-face meeting reportedly came with experience, when the focus was not on becoming familiar with the technology.

### Summary of Findings

Table 4 summarizes the findings based on the theme for each category and what disturbance caused what limitation. The text in parentheses denotes whether the disturbances and limitations were interpreted as positive or negative.

## Discussion

### Principal Findings

Our results show that a number of disturbances and perceived limitations related to the technology and surroundings occurred during video visits. These, in turn, affected how both the clinicians acted before and during video visits and the content of the visits themselves. Disturbances and perceived limitations were added to the healthcare consultation when using video communication. These disturbances and perceived limitations were caused by the technology and the patients’ and clinicians’ locations during the consultation (cf [8]). Our findings about disturbances and limitations that were related to technology addressed time (aspects of flexibility, being late or on time, waiting and logging in) and senses (hearing and seeing). The disturbances they experienced were mostly perceived as negative by the clinicians, and the limitations were mostly seen as disadvantages. However, there were some exceptions. For example, clinicians perceived video visits as easier to schedule, and the limited field of view affected the behavior of the clinician (eg, being relaxed enough not to wear shoes or to have a cup of coffee on the table). The situations we observed may not occur frequently, but they all illustrate situations that should be considered, since they may happen. In the following sections, we expand upon some features of our study to connect with topics that have been less thoroughly addressed in the literature: responsibilities that change due to a new place and space, location-related consequences, and how video visits can be improved. Our findings are in line with the “key recommendations” given by Greenhalgh et al. [8], for example, clinicians need time to develop new tasks and changes in work processes that come with a new concept. Further, new support processes and roles need to be developed and established, and there is an ongoing need to understand patients’ conditions and need for flexibility.

### From One Place to Another—Transformation of Responsibility

From our results, we can see that place still matters when using telemedicine solutions (cf [10]). The choice of place and environment for conducting video visits is important, especially as non-medical settings are added to the clinical environment (cf [15]). A video visit extends the clinical space and brings new context to a consultation, a context that leads to assumptions when a situation is interpreted. The disturbances and limitations experienced generate knowledge about what is needed to avoid them, thereby creating new requirements. The process of selecting patients for video visits and the criteria used in that process (Sturesson and Groth, in preparation) can be understood as actions to minimize the risk of disturbances and limitations in advance. Thus, to avoid disturbances and limitations, the clinicians develop requirements for the patient’s environment, or rather for the patient’s space, wanting this to be secure and comfortable for the patient (cf [8,12]). Since a video visit involves social interaction between people in different contexts, mediated by video technology, disturbances and limitations cannot always be avoided.

Both the place and the patient’s feelings in that place are important. As long as the patient feels secure and comfortable with the situation, and as long as the clinician interprets the situation as such, the patient can be at home, at work, in school, outside, at the library, in a car, or at a bus stop. Therefore, the place itself may not be a problem, as long as the risk of interruption can be minimized. However, choosing a place outside may introduce disturbances and limitations to the meeting (eg, due to background noise that might affect the ability to hear or cause other unpredictable interruptions). Patients who are new to video visits may not be aware of how their choice of space may influence the meeting. Clinicians can guide their patients in choosing a place, but the final responsibility lies in the hands of the patient. We have observed patients who were in the schoolyard, in a car, and at home in the kitchen or the bedroom. Hence, the environment and specific place where the video visit is conducted is complex and is affected by many factors that need to be accounted for (cf [13]). We have observed and described a number of situations in which the place selected by the patient caused disturbances and limitations. It is difficult for the clinician to control the patient’s space, but they can inform the patient of the requirements that need to be fulfilled to ensure the best outcome for the consultation.

There is a responsibility to select and provide a secure and comfortable environment in which the video visit can be conducted. In traditional face-to-face meetings, this responsibility is entirely the clinician’s. However, introducing video visits transfers part of this responsibility to the patient, that is, to choose a space in an environment where the patient can feel safe and comfortable for the purpose of meeting. Video visits also introduce new environmental consideration, not only in terms of being able to see and hear each other, but also what may happen in those places.

The clinicians video visit experience are aware of what can happen if the patient has not selected an optimal place for the video visit, and they can offer advice before a patient uses video visits for the first time. Clinicians who are only starting to use video visits as an alternative to physical meetings may be occupied getting everything to work, focusing on the technology, and may need support on this matter (eg, through check lists; cf [8]).

### Consequences of the Choice of Place

The video visits take place in a specific situation during a treatment program, and every visit can be seen as a social interaction between the clinician and the patient. The care is patient-centered and the meetings are based on topics the patient selects. During a meeting, the clinician thus not only adapts the content of the video visit to the patient but also to other external circumstances, that is, parts of what is happening that are not planned in advance (cf [18]). Therefore, the treatment sessions cannot be planned in detail. Using video communication adds new circumstances that can affect the content of the meeting (cf [5]). This complexity makes it important to minimize the risk of disturbances and interruptions, which may affect the meeting in a negative way.

The place, or rather what is happening in the place, that the patient has chosen can have a significant effect on the meeting and the treatment program (cf [11,13,15]). The clinician can only see parts of the place from which the patient participates. That is, for the clinician, the place includes one visible and one invisible space. Even though the patient has a responsibility for the chosen place, the clinician is still responsible for adjusting the meeting content to the selected space to meet the requirements of the environment. This requires sensitivity from the clinician’s side as well as trust in the patient’s judgment to avoid negative disturbances in terms of unaccounted limitations.

The limitations of the invisible space at the clinician’s location may be an advantage for the clinician. The physical place or the room used for video visits does not have to be designed for patient visits. Only the space visible to the video camera needs to provide a professional clinical environment. Hence, there are fewer requirements for the environment that is not part of the meeting space. In addition, the opportunity to see the environment and context of the patient’s life was considered a positive consequence of video visits because it can provide insights into the patient’s personality and the feeling of gaining a closer contact with the patient. However, this may also affect the patient in making his or her home less of a private sphere [10].

### Improvements in the Concept of Video Visits

Implementing video visits in outpatient care requires new ways of working (eg, developing criteria for patient selection). Also, new tasks are introduced when using new technologies (eg, for video communication), and a new division of labor is introduced (eg, when responsibility for one of the locations used is transferred to the patient). These changes threaten and disrupt spatial, professional, and organizational orders in the work and organization (cf [5]). These changes also introduce disturbances and limitations through both the technology (cf [5]) and surroundings (cf [8]), which need to be addressed and managed through an interplay between the technology and locations used during the video visits and the participants in the meeting.

#### Implications of Flexibility in Time and Space

In general, video visits, in comparison to face-to-face meetings, provide flexibility in time and space. This flexibility means that video visits may increase clinicians’ power over their own working time, giving them more control over their working situation. In the long run, this could reduce stress. However, video visits may also lead to stress if the visits are not easy to carry out and can thus lead to disturbances and limitations. Our analysis showed that being late caused an in situ disturbance and a perceived limitation for the clinician of not being able to communicate the delay to the patient, since there was no process or functionality for this. If the patient had been physically at the clinic, a receptionist or secretary would probably have been able to inform the patient about the delay. The feeling of not being able to notify the patient was stressful and frustrating for the clinician. The clinicians also sensed frustration from their patients in that specific situation. Hence, the virtual meeting place lacks functionality that is usually available in a physical meeting place, such as a meeting room with all its services. The place itself is important and affects the satisfaction, attitudes, and work performance of all the participants, not only the patient (cf [15]).

The awareness of being late or being on time is reciprocal. When the patient was not logged in at the scheduled time, this created uncertainty regarding if the patient had forgotten the video visit, the patient had not received the credentials to access the virtual room, or the patient was experiencing problems with technology. This uncertainty may have increased the need for the clinician to be on time and thus reduced temporal flexibility. Logging in earlier imposed a limitation on performing other duties, which in some cases led to feelings of stress and restlessness. The clinicians who were comfortable with the technology and with video visits, and who knew that their patients could also operate the technology, preferred to log in only a couple of minutes before the scheduled time of the video visit. Thus, temporal flexibility increased as participants became more comfortable with the concept.

Flexibility of place is more obvious, but has different dimensions. While logged in and waiting for the patient to log in, the clinician’s spatial flexibility and opportunities to engage in other tasks are reduced to the space captured by the video conferencing system. To notice when the patient logs in, the clinician needs to pay attention to the video conferencing window on the screen, which limits the clinician’s ability to engage in other tasks. Such limitations can be reduced if the clinician is experienced and is using his or her own office, making it possible to complete other tasks while waiting. Technical functionality could further reduce these limitations, for example, by sending a notification when the patient has logged in.

Flexibility of time and place is hence relative and exhibits different dimensions that relate to the clinician’s experiences while using video visits. This therefore affected the selection criteria used when choosing patients who are considered suitable for video visits (Sturesson and Groth, in preparation). This also needs to be considered when developing tools for, or the process of, performing video visits.

#### Implications of Issues Related to Usability of Audio and Video

Even when the clinician and patient were both logged in on time, difficulties with managing the technology, which were not unusual, could delay or otherwise affect the start of the consultation session (cf [1]). When these disturbances occurred, the clinician had to guide the patient to turn on the video or audio. Sometimes, the clinician had to guide the patient on how to log in as well. Hence, the clinician automatically took on the responsibility for first-line support, which also required that he or she had sufficient skills to guide the patient in overcoming obstacles, which were caused either by not reading the instructions properly or by the need for intricate user functionality, due to using an off-the-shelf product adapted to hospital security issues.

Disturbances and limitations, whether known or unknown beforehand, also affected the expectations and experiences of the video visit, for both the clinician and the patient. This in turn affected the content of the meeting. Anticipated disturbances and limitations were based on the clinician’s own experiences or on their colleagues’ thoughts and experience. Both anticipated and unanticipated disturbances and limitations affected the clinician’s behavior and feelings, and the content of the meeting and how the clinicians related to the different components of the concept: the technology, his or her role, the patient and his or her role, the surroundings, and the content of the treatment. Through experience, clinicians developed know-how that they were able to take to the next video visit and share with their colleagues. This experience was also used when developing selection criteria to include or exclude patients for video visits. The same know-how should be used to develop advice for the patient to use when he or she selects the place for the meeting (see above about the transformation of responsibility). Clinicians could provide this as a hand-out when offering video visits to patients.

Know-how about disturbances and limitations made it possible to adapt the video visit in advance to the technical and practical circumstances and changed personal expectations about the video visit and its outcome. Adapting the video visit to known circumstances is usually based on an individual’s or colleague’s experience of sudden disturbances; for example, knowing that audio will occasionally fail, clinicians always take their mobile phones to the meetings.

### Implications for Further Research

Further research is required to understand the full effects of video visits. Our study provides one piece of the puzzle and can guide other researchers in studying the disturbances and limitations of the digital transformation in similar or other settings. The transformation of responsibility and a focus on patient empowerment are interesting topics in today’s digital world that need further exploration. Another area that may be relevant to study, which is not addressed in this paper, is ergonomics, especially when the consultations are more complex, with a need for more detailed information.

## Abbreviations

ICT: information and communication technology
